# Phytochemical Screening, GC-MS Analysis, and Evaluating In Vivo Antitrypanosomal Effects of a Methanolic Extract of *Garcinia kola* Nuts on Rats

**DOI:** 10.3390/antibiotics12040713

**Published:** 2023-04-06

**Authors:** Fatihu Ahmad Rufa’i, Daniel Baecker, Muhammad Dauda Mukhtar

**Affiliations:** 1 Department of Microbiology, Faculty of Life Sciences, Bayero University, Kano PMB 3011, Nigeria; mdmukhtar.mcb@buk.edu.ng; 2 Kano Liaison Office, Nigerian Institute for Trypanosomiasis (and Onchocerciasis) Research, Kaduna PMB 2077, Nigeria; 3 Department of Pharmaceutical and Medicinal Chemistry, Institute of Pharmacy, University of Greifswald, Friedrich-Ludwig-Jahn-Straße 17, 17489 Greifswald, Germany; daniel.baecker@uni-greifswald.de

**Keywords:** *Garcinia kola*, methanolic extract, sleeping sickness, suramin, *Trypanosoma brucei brucei*, trypanosomiasis

## Abstract

Trypanosomiasis is a serious disease that affects both humans and animals, causing social and economic losses. Efforts to find new therapeutic approaches are warranted to improve treatment options. Therefore, the purpose of this communication includes the phytochemical screening of a methanolic extract of *Garcinia kola* nuts and the in vivo evaluation of its biological activity against rats infected with *Trypanosoma brucei brucei* and treated with 4 different concentrations of the extract (0.01, 0.1, 1, and 10 mg/kg). Treatment with suramin served as a positive control, while the negative control received no drug. Since the general toxicity of the extract could be ruled out, efficacy was evaluated based on physiological changes, such as induction of trypanosome parasitemia, influence on body temperature, and body weight. Survival was assessed during this study. Physical parameters, behavioral characteristics, and various hematological indices were also monitored. Based on the (patho)physiological and behavioral parameters (e.g., no parasitemia, no increase in body temperature, an increase in body weight, no loss of condition, no alopecia, and no gangrene), the efficacy of the extract was evident, which was also confirmed by 100% survival, while in the negative control, all rats died during the observation period. Since overall very similar results were obtained as a result of treatment with the established suramin, the in vivo antitrypanosomal activity of a methanolic extract of *G. kola* nuts on rats can be demonstrated in this communication. This opens the way, for example, for further development of drug formulations based on this methanolic extract.

## 1. Introduction

Trypanosomiasis is a diverse and deadly disease that affects both humans and animals [1]. In humans, it is known as “sleeping sickness,” while in domestic animals, it is called “samore” or “nagana” [2]. It is caused by African trypanosome species, including *Trypanosoma brucei brucei*, *T. congolense*, *T. vivax*, *T. evansi*, and *T. equiperdum*. The pathogens are transmitted by blood-sucking insects, the tsetse flies [2]. These flies are found in the tropical region of Africa, where they invest 11 million square kilometers and are distributed across 36 sub-Saharan countries [3]. Approximately 8.6 million cattle and more than 2.6 million people are at risk of infection [1], making the disease a priority tropical disease due to its high number of deaths and economic losses [3].

The drug suramin has long been used to treat trypanosomiasis, but only in the first stage of sleeping sickness [4]. In addition, the suramin suffers from a variety of side effects to further limit its use. Therefore, in principle, the World Health Organization (WHO) recommends vigorous efforts for a continuous search for safer, effective, and affordable medicines to achieve effective control and eradication of the threat [5], especially preparations from African medicinal plants have promising potential to act as antiprotozoal agents [6,7]. These include, for example, *Garcinia kola* due to its pharmacognostic properties as a source of antitrypanosomal drug candidates [8].

*Garcinia kola* is a dicotyledonous flowering plant belonging to the *Clusiaceae* family [9]. The genus contains only one species *G. kola*. It is distributed in West Africa, particularly in Nigeria [10]. Its trees grow in rainforests of medium size, up to 12–14 m tall, and produce reddish-yellow or orange fruits. Each fruit contains 2–4 yellow nuts and a sour-tasting pulp. The nuts are commonly called bitter kola because of their bitter astringent taste [11,12]. *G. kola* has been identified as a potent antibiotic [9]. It is one of the plants commonly used for its nutritional value and medicinal properties. All parts of this plant, including the nut, leaf, stem bark, and root, have been mentioned in many ethnobotanical and pharmacological studies. Extracts from different parts of *G. kola* are used extensively in African traditional medicine [13] and show promise in treating many different diseases [9,11,14,15]. However, the nut seems to be the most commonly used part of the plant.

Previous research has focused on the antitrypanosomal properties of an ethanolic leaf extract of *G. kola* [9]. Elsewhere, the trypanocidal properties of various fractions of an ethanolic seed extract have been determined, with the authors suggesting in vivo studies to further verify the potential of *G. kola* [16]. However, initial in vivo studies with a methanolic extract of *G. kola* nuts were conducted using mice as experimental animals [11]. In contrast to mice, rats are superior, especially in terms of cognitive behavior. To our best knowledge, studies on rats that investigate a methanolic extract of *G. kola* nuts are lacking in the literature so far. We chose a methanolic extract for our study for two reasons. First, the methanolic extract of *G. kola* has a better extractive yield of bioactive ingredients compared to the ethanolic extract [17]. On the other hand, methanol (65 °C) as a solvent has a somewhat lower boiling point than ethanol (78 °C), which makes it easier and somewhat gentler to evaporate the solvent after the extraction process.

In this communication, a methanolic extract of *G. kola* nuts was assessed for its phytochemical constituents, tested for its general toxicity against rats, and finally, the efficacy of the extract for treatment after infection with trypanosomes based on obvious parameters. In the evaluation, besides physical parameters, also behavioral changes of the test animals were considered.

## 2. Results and Discussion

### 2.1. Phytochemical Characterization

The methanolic extract of *G. kola* was screened first for common groups of compounds with respect to its content. The phytochemical detection reactions revealed the general presence of saponins, tannins, glycosides, terpenoids, steroids, alkaloids, and flavonoids. This is in agreement with a similar study by Ogbadoyi et al. [11], also describing saponins, tannins, glycosides, terpenoids, steroids, alkaloids, and flavonoids as major constituents of a methanolic extract of *G. kola* nuts.

These compounds are associated with the bioactivity of the extract. The properties of flavonoids, for instance, in *G. kola* promise great benefits. Flavonoids are generally known to be effective antioxidants and, therefore, can protect cells from the harmful effects of reactive oxygen species (ROS). These may even lead to anemia, which is characterized by a significant reduction in red blood cells and is one of the major pathological effects of trypanosome infection [16]. However, flavonoids can counteract the destructive imbalance of ROS. In addition, the antitrypanosomal property of alkaloids is attributed to interaction with DNA associated with inhibition of protein biosynthesis. The antitrypanosomal activity of saponins could be the result of their general cytotoxicity [11].

In addition to phytochemical assays, gas chromatography mass spectrometry (GC-MS) is a useful method to obtain a somewhat clearer picture of the pharmaceutical ingredients and has, therefore, also been applied to preparations of *G. kola* [18]. Thus, GC-MS analysis is considered the first step to better understanding the chemical constituents present in certain plants. Hence, such analysis was performed adducing the current methanolic extract of *G. kola* nuts.

Table 1 summarizes the retention times of the chemical compounds present in the extract and their percentage area of GC-MS analysis. Tridecanoic acid was eluted first with a retention time (t_R_) of 14.69 min and a percentage area under the cure (AUC) of 4.01%, then cyclohexane with t_R_ = 17.48 min and AUC = 2.86%, followed by hexanedioic (adipic) acid with t_R_ = 22.22 min (AUC = 45.3%), benzene (t_R_ = 22.58 min, AUC = 3.60%), tetrasiloxane (t_R_ = 27.76 min, AUC = 15.4%), thymol (t_R_ = 28.04 min, AUC = 3.88%), pentanone (t_R_ = 28.44 min, AUC = 6.06%), silane (t_R_ = 28.59 min, AUC = 2.37%), and phenol (t_R_ = 29.02 min, AUC = 2.02%).

The compounds identified in the methanolic extract are believed to have different biological activities for medicinal purposes. Due to its, by far, highest occurring content, hexanedioic (adipic) acid seems to be the active ingredient. Therefore, the antitrypanosomal, antimicrobial, and antioxidant activities reported for hexanedioic (adipic) acid may provide the rationale for the traditional use of *G. kola* [19].

Moreover, the compounds benzene (and especially derivatives thereof) and hexanedioic (adipic) acid are known to exhibit a variety of pharmaceutical values, such as antioxidative, anti-inflammatory, antimicrobial, antiparasitic effects, and potent antitrypanosomal activity [20,21,22,23,24]. The antiprotozoal activity of thymol was also reported before [25].

### 2.2. General Toxicity and Antitrypanosomal Activity

The acute total toxicity of the methanolic extract of *G. kola* nuts was studied by treating albino rats with increasing concentrations of the extract and considering the potential lethality. The results of this experimental approach are summarized in Table 2.

None of the animals died within 24 h after oral administration of the methanolic extract at a dose up to 5000 mg/kg body weight. This indicates that the methanolic extract of *G. kola* is relatively non-toxic to rats. Moreover, the results are in agreement with the work of Sani et al. [9], which proved the ethanolic leaf extract of *G. kola* to be a safe and effective herbal medicine.

**Figure 1:**
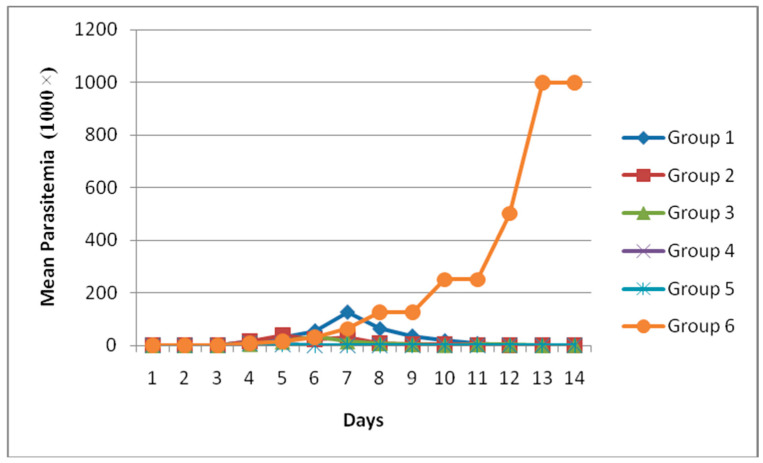
Mean (*n* = 2) trypanosome parasitemia of the experimental animals within 14 days.

Parasitemia describes the number of parasites in the blood and is, therefore, a suitable parameter to express the antitrypanosomal efficacy of a drug after infection with pathogens [26]. The mean parasitemia of the experimental animals infected with *T. b. brucei* is presented in Figure 1.

In all 6 groups (2 rats each), parasitemia became visible microscopically 4 days after infection, while daily treatment was also started immediately. In group 6 (negative control, without any treatment), parasitemia increased throughout the experimental period. A slight increase in parasitemia was also observed in group 1 (lowest test concentration of 0.01 mg/kg) until the 7th day. However, thereafter, the decrease occurred again. Presumably, the dose seems capable of increasing for an immediate antitryanosomal effect. Such a dose–response relationship can be seen in the results of the groups with higher concentration treatment. Indeed, very little or no parasitemia was observed in group 2 (0.1 mg/kg) and group 3 (1 mg/kg). Moreover, the complete disappearance of parasitemia was documented in group 4 (10 mg/kg) and also in group 5 (positive control, treatment with suramin). This confirms the effect of the treatment.

**Figure 2:**
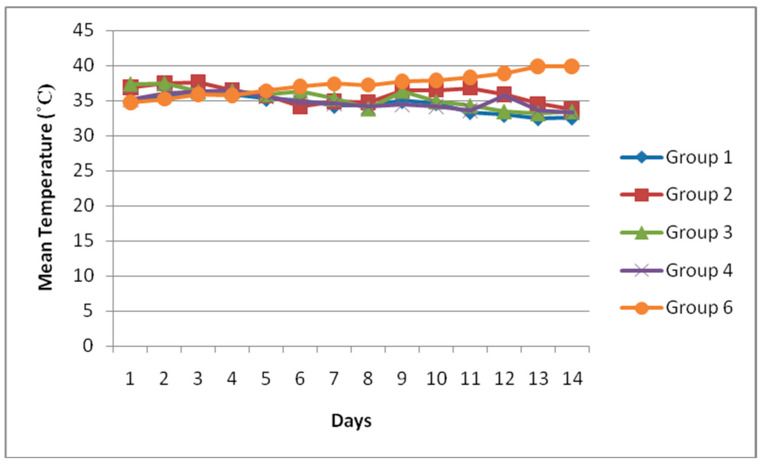
Mean (*n* = 2) temperature of the experimental animals within 14 days.

Fever is a common symptom of sleeping sickness [27]. Therefore, measuring the body temperature of the test rats is also an appropriate parameter for assessing antitrypanosomal effects. The mean temperature was measured over the 14 days test period, and the results are shown in Figure 2.

In group 6 (negative control), the mean body temperature increased continuously up to 40 °C on the 14th day after infection. Group 1 (0.01 mg/kg), group 2 (0.1 mg/kg), group 3 (1 mg/kg), group 4 (10 mg/kg), and group 5 (positive control, treated with suramin) nearly maintained a body temperature of (36–37 °C) from day 1–3, after which the temperature decreased to (34–36 °C) on day 14 after infection. Compared to the negative group, these results indicate an impact on the treatment. 

**Figure 3:**
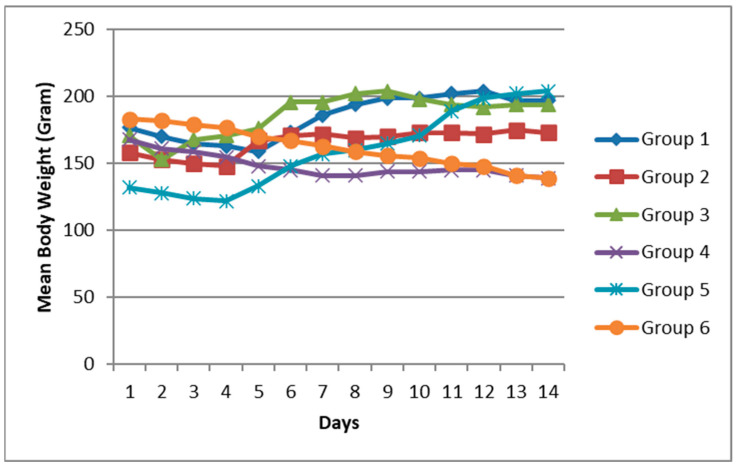
Mean (*n* = 2) body weight of the experimental animals within 14 days.

Changes in body weight were also found as a symptom in rats following infection with *T. b. brucei* [28], and thus, it was monitored in this current study (Figure 3).

During the first 4 days after infection, i.e., until parasitemia was detected by microscopic examination and no treatment was administered yet, all 6 groups showed a slight decrease in body mass, although this fluctuated somewhat in group 3. After day 4, the treatment was carried out according to the protocol until day 14. The rats treated with the extract showed an increase in body mass until the end of the observation period, except group 4, which received the extract at the highest concentration. Strikingly, the rats treated with the positive control suramin (group 5) showed the relatively greatest increase in body mass. There are different data in the literature regarding the effect of suramin on this [29,30]. However, more importantly, group 6 (negative control), which did not receive any treatment, showed a continuous decrease in body weight over the 14 days observation period. Compared to the drug-treated rats, an indication of the onset of the effect can be deduced.

**Figure 4:**
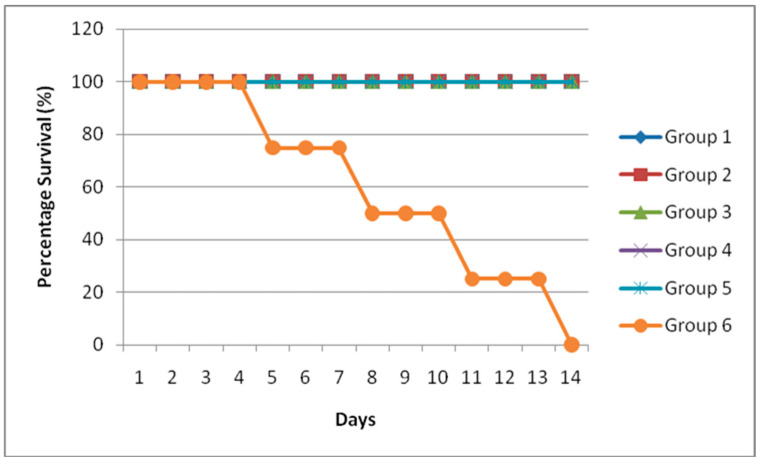
Percentage of survival of the experimental animals within 14 days.

Finally, the percentage of survival after infection with *T. b. brucei* was evaluated as shown in Figure 4.

All groups 1–4 treated with the methanolic extract of *G. kola*, and the positive control (treated with suramin, group 5) had 100% survival throughout the observation period (14 days). Strikingly, a survival rate of 0% was observed in the negative control (group 6) after 14 days. These results clearly demonstrate the value of treatment with the extract of *G. kola*.

The parameters parasitemia, body temperature, and weight, as well as survival, used to characterize the disease [31] indicate the benefit of treating rats infected with *T. b. brucei* with the methanolic extract of *G. kola* nuts. Moreover, the general toxicity of the extract seems to be excluded. Nevertheless, it is important to consider further physical and behavioral changes (Table 3) of the experimental animals to evaluate the application of the extract.

In the animals treated with any concentration of the extract, loss of condition, alopecia, and gangrene were totally absent. The parameters of pallor of the mucous membrane, pyrexia, lacrimation eye redness, and ringtail were also absent or only low represented. A correlation with a rise in the extract concentration among groups 1–4 can, therefore, not be deduced. The behavior with regard to food consumption, which was described in the literature as increasing after infection [32], was somewhat different. In groups 1 (0.01 mg/kg) and 2 (0.1 mg/kg), this was moderate and even high in the higher concentrated groups 3 (1 mg/kg) and 4 (10 mg/kg). High food consumption was also present in the suramin-treated rats but was low in the untreated animals. On the other hand, it is striking that the rats treated with the highly concentrated extract (group 4, 10 mg/kg) and with the positive control suramin showed a moderate degree of aggression, which was not the case in the other groups, including the negative control (group 6). The untreated animals (group 6) showed a significant increase in the parameters of pallor of mucous, loss of condition pyrexia, and lacrimation. This corresponds to the reports of the WHO [5] characterizing trypanosomiasis.

With the exception of the increased food uptake, which can also be attributed to infection, and the moderate aggression only at the highest applied extract concentration, no negative effects on physical and behavioral behavior seem to be evident. More importantly, there was an absence of core symptoms compared to the untreated group, which is in favor of the extract.

### 2.3. Influence on Haematological Indices

As reported by Maigari and Dabo [33], anemia is a useful and common sign of trypanosomiasis, and its degree determines the severity of the infection. Therefore, different hematological indices (Table 4) describing anemia were also used for assessment in this study. These include packed cell volume (PCV), hemoglobin (Hgb), red blood cells (RBC), mean corpuscular volume (MCV), mean corpuscular hemoglobin (MCH), and mean corpuscular hemoglobin concentration (MCHC).

Compared with the positive control (group 5), the extract-treated rats (groups 1–4) showed lower PCV. With decreasing concentration of the extract, the value also became lower. For the negative control (group 6), the value was only half that of the lowest concentrated extract. When treated with the extract, Hgb was slightly decreased in the rats. A correlation with its concentration could not be deduced. The untreated rats showed remarkably low Hgb content. Regarding RBC, a slight increase with rising extract concentration could be assumed. However, the mean value is comparable to the positive control. In group 6, RBC was significantly decreased. Concerning MCV and MCH, the values of the extracts are comparable to the suramin-treated group but significantly lower than the untreated group. Regarding MCHC, there were hardly any meaningful differences among all groups 1–6. However, obvious signs of anemia can be excluded from these results after extract treatment.

In group 6, which did not receive any drug, strongly deviating parameters were noticeable. Due to the lack of treatment, there may be increased damage to red blood cells, and the movement of the parasite in the bloodstream leads to obstruction and removal of blood cells by the expanding mononuclear phagocytic system, resulting in anemia. Therefore, this can be used as a useful indicator of trypanosomiasis [33] as applied herein.

## 3. Materials and Methods

### 3.1. Chemicals and Reagents

All the chemicals and reagents used throughout this study were obtained from registered vendors.

### 3.2. Plant Collection, Authentication, Preparation, and Extraction Procedure

Fresh nuts of *Garcinia kola* were purchased from an appropriate outlet at the Sabon Gari market in Kano, Nigeria. The acquired nuts were then authenticated from the herbarium of the Department of Plant Sciences, Bayero University, Kano, Nigeria, based on the established botanical criteria of the herbarium.

After washing with water, the nuts were crushed using a hand grinder and then dried at room temperature for 7 days in a plastic container. The dried product was pounded into a fine powder using a mortar and pestle and then stored in a dry and sterile container to avoid contamination by environmental pathogens.

The powder was extracted by cold maceration with methanol. The amount of 300 g of the powder was weighed and dissolved in 900 mL of methanol, resulting in a ratio of 1:3 of plant product to solvent. This mixture was then allowed to stand for 2 days with regular shaking, followed by filtering through a muslin cloth and evaporation of the solvent in a water bath at 40 °C. The same methanolic extract was applied during this study.

### 3.3. Identification of Constituents

The phytochemical constituents of the extract were determined according to the standard screening method of Silva et al. [34] and analyzed concerning the general presence of saponins, tannins, glycosides, terpenoids, steroids, alkaloids, and flavonoids. The experimental procedure of the phytochemical screening is briefly explained below in each case:

Test for saponins: 10 mL of distilled water was added to a test tube containing 1 g of the extract and shaken vigorously. The presence of persistent foam indicated the presence of saponins.

Test for tannins: 10 mL of distilled water was mixed with 1 g of the extract and heated in a water bath (at 40 °C). The mixture was filtered, and a solution of ferric chloride (0.5%) was added. The presence of a dark green or blue-black color indicated the presence of tannins.

Test for glycosides: 10 mL of distilled water was added to a test tube containing 1 g of the extract and shaken vigorously. 10 mL of ferric chloride (0.5%) and 1 mL of glacial acetic acid were added. The presence of a brown ring indicated the presence of glycosides.

Test for terpenoids: 10 mL of distilled water was added to a test tube containing 1 g of the extract and shaken vigorously. 10 mL of ferric chloride (0.5%) and 1 mL of concentrated sulfuric acid were added. A reddish-brown coloration indicated the presence of terpenoids.

Test for steroids: 10 mL of distilled water was added to a test tube containing 1 g of the extract, shaken vigorously, and mixed with 1 mL of concentrated sulfuric acid. The occurrence of a reddish-brown color indicated the presence of steroids.

Test for alkaloids: A few drops of diluted hydrochloric acid and Dragendorff reagent were added to 10 mL of distilled water in a test tube containing 1 g of extract and shaken vigorously. The presence of a red or pink precipitate indicated the presence of alkaloids.

Test for flavonoids: 2 mL of a solution of sodium hydroxide (2.0%) was added to a test tube containing 1 g of extract and 10 mL of distilled water, and the mixture was shaken vigorously. The occurrence of a yellow color indicated the presence of flavonoids.

In addition, the powdered form of the extract was reconstituted in methanol and subject to a GC-MS analysis in order to determine its chemical constituents. For this analysis, a gas chromatography system (containing gas cylinder, flow regulator, and injection port) and a quadrupole mass spectrometer both manufactured by Biobase (Jinan, China) were used.

### 3.4. Experimental Animals and Parasites

A total of 12 adult male and female albino rats, approximately 7 weeks of age and weighing 140–200 g each, were used in this study. They were obtained from the animal house of the Department of Human Anatomy, Bayero University, Kano, Nigeria. The rats were allowed to acclimate for 14 days in the research laboratory where the investigations were performed. They were housed in plastic cages under standard hygienic conditions, fed commercial feed (Vital feeds LTD, Kano, Nigeria), and had ad libitum access to clean water appropriately maintained by the animal keeper.

*Trypanosoma brucei brucei* (Federi strain) obtained from the Nigerian Institute for Trypanosomiasis and Onchocerciasis Research, Vom, Plateau State, Nigeria, was used for this study. The parasite was first isolated from cattle in 2018, identified as *T. b. brucei*, and stabilized by four passages in rats before storage in liquid nitrogen. It was confirmed and characterized using the standard trypanosome detection procedures of Woo [35] as described by Nakayima [36]. The parasite was maintained by serial passages in donor rats, then the infected blood of the donor rat was collected at the peak of parasitemia by tail bleeding and diluted with physiological saline, which was inoculated into the peritoneal cavity of the experimental rats.

All experimental protocols were conducted similarly to a study published earlier by our group [37] and in strict compliance with the guidelines for the care and use of animals established by the Ethics Committee of Bayero University, Kano, Nigeria. This study has received ethical approval (protocol code: 21.01.13.1231/TR and date: 12 January 2021).

### 3.5. Determination of Acute Lethal Toxicity

The acute lethal toxicity of the methanolic extract of *Garcinia kola* was screened according to the established method by Lorke [38]. A total of 12 albino rats were used for both the first and the second experiments. In the first series, 9 rats were randomly divided into 3 groups of 3 animals each and orally treated with the extract at a dose of 10 mg/kg, 100 mg/kg, and 1000 mg/kg, respectively. The second series was performed with another group of 3 rats randomly divided into 3 groups of 1 animal each and treated orally with 1600 mg/kg, 2900 mg/kg, and 5000 mg/kg, respectively. The mortality rate 24 h after treatment was assessed as outcome.

### 3.6. Experimental Design and Inoculation with Trypanosomes

A total of 12 experimental rats (different than that of the toxicity testing) were randomly divided into 6 groups (1–6) of 2 animals each. All groups (1–6) were infected intraperitoneally with 0.1 mL of blood containing approximately 10^6^ trypanosomes/mL. Treatment began on the day the parasites were first detected microscopically in the bloodstream (day 4) and continued until day 14 when the entire negative control died. Groups 1–4 received the methanolic extract of *Garcinia kola* intraperitoneally at daily doses of 0.01, 0.1, 1.0, and 10 mg//kg body weight, respectively. Group 5 served as a positive control and received the standard trypanosome drug suramin of 7.86 mg/kg. Group 6 was infected without any treatment (negative control).

### 3.7. Monitoring Different Outcome Parameters

Parasitemia was monitored daily using the rapid matching method of Herbert and Lumsden [26] by preparing a wet preparation from peripheral blood by tail bleeding, in which a drop of blood was placed on a clean slide and covered with coverslip. Each slide was prepared separately and observed. The number of parasites was determined microscopically by counting the parasites in each field and comparing them with a standard.

The body temperature of all experimental animals was measured daily. Each rat was gently trapped, and a digital thermometer (Biobase, Jinan, China) was inserted 3 cm into the anus of each rat upon hearing a beep, the thermometer was immediately withdrawn, and the values obtained were recorded as described by Ajakaiye et al. [39].

Daily body weight of the experimental rats was determined using a digital scale (Biobase, Jinan, China).

The percentage of survival was monitored daily and expressed as the number of survivors divided by the total original number in the group multiplied by 100% [37].

Physical and behavioral changes, such as mucosal condition, loss of condition, pyrexia, lacrimation, food intake, eye redness, and aggression, among others, were observed and recorded during the experiments. The classification into the different severity levels was conducted according to the grades high, moderate, low, and absent, respectively.

Hematological indices, such as packed cell volume (PCV), red blood cells (RBC), hemoglobin concentration (Hbg), mean corpuscular volume (MCV), mean corpuscular hemoglobin (MCH), and mean corpuscular hemoglobin concentration (MCHC), were all determined using an automated hematological blood analyzer (Biobase, Jinan, China) [33].

### 3.8. Data Analysis

All data were expressed as mean with standard error. They were analyzed using the statistical software SPSS version 20, 1-way ANOVA test at a degree of freedom of 0.05.

## 4. Conclusions

Due to the social and economic importance of trypanosomiasis, especially on the African continent, research for antiprotozoal treatments is urgently indicated. For this purpose, traditionally used indigenous plants such as *Garcinia kola* show promising potential. In this communication, the methanolic extract of *G. kola* nuts was characterized in terms of phytochemical composition, with hexanedioic (adipic) acid considered the compound mainly responsible for the biological effect. Since the extracts did not show general toxicity in an initial screening, in vivo studies with rats were performed. As a result of the treatment of experimental animals infected with *T. b. brucei*, there was no trypanosome parasitemia, no increase in body temperature, no decrease in body weight, and complete survival during the observation period. This contrasted with the untreated group. In addition, it was focused on hematological, physical, and behavioral changes upon application of the extract. Based on the data, anemia can be excluded by comparison with the negative control. With the exception of moderate aggression with the highest concentrated extract, which also occurred in the rat treated with the established drug suramin, there were no significant changes. A limitation of this present study is the small number of experimental animals, which, however, should be sufficient to get a first insight into the antitrypanosomal effects reported in this communication.

In this small study, the in vivo antitrypanosomal effects of the methanolic extract of *G. kola* nuts were also demonstrated in rats, paving the way for future in-depth research. This may refer, for example, to focusing on specific fractions of the extract, isolating, and elucidating the main molecules, until finally referring to an eventual formulation of antitrypanosomal drugs based on the methanolic extract.

## Figures and Tables

**Table 1:** Results from the GC-MS analysis of the methanolic extract of *G. kola*.

Chemical Compound	Retention Time (min)	Percentage Area (%)
Tridecanoic acid	14.69	4.01
Cyclohexane	17.48	2.86
Hexanedioic (adipic) acid	22.22	45.3
Benzene	22.58	3.60
Tetrasiloxane	27.76	15.4
Thymol	28.04	3.88
Pentanone	28.44	6.06
Silane	28.59	2.37
Phenol	29.02	2.02

**Table 2:** Acute toxicity after orally administering the crude methanolic extract (CME) of *G. kola* to the experimental rats.

Experiment	Dose/Body Weight (mg/kg)	Number of Animals Used	Number of Animals Dead
Phase 1	10	3	0
	100	3	0
	1000	3	0
Phase 2	1600	1	0
	2900	1	0
	5000	1	0

**Table 3:** Physical and behavioral changes of experimental animals.

Parameter ^1^	Group 1	Group 2	Group 3	Group 4	Group 5	Group 6
Pallor of mucous membrane	+	-	-	-	-	+++
Loss of condition	-	-	-	-	-	+++
Pyrexia	-	-	+	-	-	+++
Lacrimation	-	+	-	-	-	+++
Eye redness	+	-	-	+	+	+
Aggression	-	-	-	++	++	-
Alopecia	-	-	-	-	-	-
Ringtail	-	+	-	-	-	-
Gangrene	-	-	-	-	-	-
Food consumption	++	++	+++	+++	+++	+

^1^ Assessment according to the different degrees high (+++), moderate (++), low (+), and absent (-).

**Table 4:** Hematological indices of experimental animals. The data represent the mean ± standard error of *n* = 6 independent experiments.

Parameter	Group 1	Group 2	Group 3	Group 4	Group 5	Group 6	*p* Value ^1^
PCV (%)	28.5 ± 1.6	30.0 ± 0.0	32.8 ± 1.0	33.8 ± 0.3	36.0 ± 0.6	14.5 ± 0.3	<0.001
Hgb (g/dL)	9.65 ± 0.59	12.9 ± 2.4	11.1 ± 0.3	11.2 ± 0.1	12.2 ± 0.1	4.70 ± 0.06	<0.001
RBC (10^6^/μL)	4.73 ± 0.11	4.85 ± 0.05	5.98 ± 0.10	5.18 ± 0.03	5.40 ± 0.14	2.85 ± 0.03	<0.001
MCV (fl)	60.2 ± 1.9	62.9 ± 0.4	64.5 ± 0.7	63.7 ± 0.4	65.5 ± 0.4	50.8 ± 0.5	<0.001
MCH (pg)	20.4 ± 0.8	21.5 ± 0.2	21.8 ± 0.2	21.3 ± 0.1	22.1 ± 0.0	16.5 ± 0.1	<0.001
MCHC (g/dL)	33.8 ± 0.2	34.1 ± 0.1	33.7 ± 0.1	33.8 ± 0.2	33.7 ± 0.1	32.4 ± 0.2	<0.001

^1^ The *p* value is based on the comparison to group 5 (positive control).

## Data Availability

Data is contained within the article.
